# Carvacrol prevents acrylamide-induced oxidative and inflammatory liver damage and dysfunction in rats

**DOI:** 10.3389/fphar.2023.1161448

**Published:** 2023-04-05

**Authors:** Serkan Cerrah, Fatih Ozcicek, Betul Gundogdu, Betul Cicek, Taha Abdulkadir Coban, Bahadir Suleyman, Durdu Altuner, Seval Bulut, Halis Suleyman

**Affiliations:** ^1^ Division of Gastroenterology, Department of Internal Medicine, Erzurum Regional Training and Research Hospital, Erzurum, Türkiye; ^2^ Department of Internal Medicine, Faculty of Medicine, Erzincan Binali Yildirim University, Erzincan, Türkiye; ^3^ Department of Pathology, Faculty of Medicine, Ataturk University, Erzurum, Türkiye; ^4^ Department of Physiology, Faculty of Medicine, Erzincan Binali Yildirim University, Erzincan, Türkiye; ^5^ Department of Clinical Biochemistry, Faculty of Medicine, Erzincan Binali Yildirim University, Erzincan, Türkiye; ^6^ Department of Pharmacology, Faculty of Medicine, Erzincan Binali Yildirim University, Erzincan, Türkiye

**Keywords:** carvacrol, experimental models, liver damage, oxidative stress, acrylamide

## Abstract

**Background:** Acrylamide causes hepatotoxicity with the effect of oxidative stress and inflammatory processes. Carvacrol is a monoterpenic phenol with antioxidant and anti-inflammatory properties.

**Aims:** To determine the effects of carvacrol on oxidative liver injury induced by acrylamide administration in rats.

**Methods:** Rats were divided into three groups of six animals each: healthy group acrylamide group (ACR), and acrylamide + carvacrol group (TACR). First, carvacrol (50 mg/kg) was administered intraperitoneally to the CACR group. One hour later, acrylamide (20 mg/kg) was given orally to the ACR and CACR groups. This procedure was performed for 30 days, after which the animals were sacrificed. The malondialdehyde (MDA) and total glutathione (tGSH) levels, total oxidant (TOS) and total antioxidant status (TAS), tumor necrosis factor-alpha (TNF-α), interleukin-1beta (IL-1β), and nuclear factor kappa b (NF-κB) were measured in the excised liver tissues. Alanine aminotransferase (ALT) and aspartate aminotransferase (AST) levels were determined in blood serum samples. Liver tissues were also examined histopathologically.

**Results:** In the ACR group, malondialdehyde, TOS, ALT, AST levels, and NF-κB, IL-1β, and TNF-α levels were found to be high, and tGSH and total antioxidant status levels were low. In addition, diffuse degenerative changes and necrosis in hepatocytes, and moderate inflammation in the portal region were detected in the liver tissues of the ACR group. While carvacrol prevented the biochemical changes induced by acrylamide, it also alleviated the damage in the histological structure.

**Conclusion:** Carvacrol may be used for liver damage caused by acrylamide.

## 1 Introduction

Acrylamide (CH2 = CHCONH2) is a colorless and odorless toxic molecule (Friedman, 2003). Acrylamide is known as a food contaminant (Mogol and Gökmen, 2016). Acrylamide is produced in foods subjected to high-temperature treatments (Rifai and Saleh, 2020). It is intaken with fried, baked, and roasted foods that are widely consumed, especially by children, teenagers, and adults (Matoso et al., 2019). Before its discovery in food, acrylamide was a chemical compound used in many industrial processes such as plastics, adhesives, and paper production (Mogol and Gökmen, 2016). It has been reported that acrylamide exposure can cause various pathologies in humans (LoPachin and Gavin, 2012). Cigarette smoke is one of the main causes of acrylamide exposure (Ibrahim and Ibrahem, 2020). Neurotoxicity, cardiotoxicity, and hepatotoxicity are acrylamide-related toxicities (Kunnel et al., 2019).

It is mentioned in the literature that acrylamide causes the release of large amounts of reactive oxygen species (ROS) and oxidative stress (OS) (Hong et al., 2021). Damage caused by oxidative stress also refers to changes in signaling pathways (Hong et al., 2019). Nuclear factor erythroid 2 (Nrf2), a transcription factor and antioxidant response regulator, is affected by oxidative stress and lack of Nrf2 activation leads to hepatotoxicity. Mitogen-activated protein kinase (MAPK) phosphorylation is induced in the presence of OS and leads to cell death *via* associated apoptosis signals (Renu et al., 2020; Hong et al., 2021). The MAPK cascade consists of three major kinases: c-Jun N-terminal kinase (JNK), extracellular receptor kinase (ERK), and p38. In response to ROS production, these kinases increase mitochondrial dysfunction associated with cell damage (Renu et al., 2020).

Artan ROS seviyeleri hücresel makromolekülleri zarar verebilir (Hong et al., 2019). Erfan et al. showed that malondialdehyde (MDA), formed as a result of lipid peroxidation (LPO), is one of the important components in the pathogenesis of acrylamide hepatotoxicity. They found that acrylamide increased alanine aminotransaminase (ALT) and aspartate aminotransferase AST levels together with MDA in liver tissues, decreased SOD levels and caused tissue damage (Erfan et al., 2021). Again, in acrylamide-related hepatotoxicity, apart from oxidant and antioxidant parameters, pro-inflammatory cytokines have been reported to play a role and demonstrated that acrylamide caused an increase in the expression of interleukin-1beta (IL-1β), interleukin-6, tumor necrosis factor-alpha (TNF-α) and nuclear factor kappa b (NF- κB) in liver and kidney tissues (Kandemir et al., 2020). This literature information shows that antioxidant and anti-inflammatory drugs can be used in acrylamide hepatotoxicity.

Carvacrol, which we have planned to investigate its effect against acrylamide hepatotoxicity, is a monoterpenic phenol found in the oil of thyme and some plants (De Vincenzi et al., 2004). Although there are many animal studies on carvacrol, human studies are limited. A study evaluating the safety and tolerability effects of carvacrol in healthy subjects showed that administration of carvacrol for 1 month did not cause any major adverse effects. In addition, Carvacrol is considered a safe chemical at low concentrations and has been approved by the Federal Drug Administration (FDA) for use as a preservative in foods (Ghorani et al., 2021). Studies have confirmed that Carvacrol has antimicrobial, bactericidal, anti-inflammatory, anticancer, antioxidant, antifungal, and antidepressant properties, and biological activities as a modulator of nerve impulses, and an immunological modulator (Silva et al., 2018). It has been reported that carvacrol exhibits hepatoprotective activity by increasing the total antioxidant capacity (TAS), suppressing the increase in total oxidant capacity (TOS) and MDA (Mohseni et al., 2020). It has been reported that carvacrol has hepatoprotective properties by suppressing the expression of TNF-α, interleukin-6, and NF- κB (Aristatile et al., 2013; Alshehri and Alorfi, 2023). Again, carvacrol has been documented to prevent the increase in liver function tests with ethanol (Khan et al., 2019). This information on the biological activity of carvacrol suggests that it can be used against acrylamide-induced liver injury. Therefore, the current study, it was aimed to investigate the effect of carvacrol on acrylamide-induced liver damage and dysfunction in rats through biochemical and histopathological examination.

## 2 Materials and methods

### 2.1 Chemicals

Of the chemicals used, carvacrol was supplied from Sigma-Aldrich, Inc (Missouri, USA), thiopental sodium, I.E., Ulagay (Istanbul, Turkey), and acrylamide Sigma-Aldrich, Inc. (St. Louis, USA).

### 2.2 Animals

Eighteen male Albino Wistar rats (267–275 g) included in the study were purchased from Erzincan Binali Yıldırım University Experimental Animals Application and Research Center. The animals were housed at the appropriate temperature (21°C–23°C) in the environment where the experiment would take place, 1 week before the experiment so that they could adapt to the environment. A 12-h light-dark cycle was provided. The animals were fed *ad libitum* with normal water and feed. Experimental applications were carried out by international laboratory animal use and care guidelines (ARRIVE).

### 2.3 Experimental groups

Three groups of six animals were formed: the healthy group (HG), the acrylamide group (ACR), and the carvacrol + acrylamide group (CACR).

### 2.4 Experiment procedure

Carvacrol was administered intraperitoneally (i.p) at a dose of 50 mg/kg (Gore Karaali et al., 2022; Salmani et al., 2022) (LD50 for rats: 810 mg/kg) to the CACR group. ACR and HG groups received the same amount of distilled water (i.p) simultaneously. After these procedures, acrylamide (20 mg/kg) (Erfan et al., 2021) was given orally to the rats in the CACR and ACR groups. The HG group received the same amount of orally distilled water. These applications were continued once a day for 30 days (Karimi et al., 2020). Animals were euthanized with 50 mg/kg of thiopental sodium 24 h after the 30_th_ dose of acrylamide, and the liver tissues of all animals were removed for examination. MDA, total glutathione (tGSH), TOS, TAS, TNF-α, IL-1β, and NF-κB levels in liver tissues were measured. In addition, the histological structures of the tissues were examined. Before the animals were euthanized, blood samples were taken for the determination of ALT and AST activity by entering the tail veins of the rats. The data obtained were compared between groups.

### 2.5 Biochemical analysis

MDA measurements, Ohkawa et al. (1979) tGSH measurements were made by the method described by Sedlak and Lindsay (Sedlak and Lindsay, 1968).

TOS and TAS measurements were determined with the automatic measurement method developed by Erel and using commercial kits (Rel Assay Diagnostics, Turkey) (Erel, 2004b; Erel, 2005).

TNF-α, IL-1β and NF-κB analyses were performed using a commercial kit according to the rat-specific sandwich enzyme-linked immunosorbent assay. Rat TNF-α and Rat IL-1β ELISA kits (Cat no: YHB1098Ra, Shanghai LZ); NF-κB ELISA kits (Cat. No: 201-11-0288, SunRed). The examinations were carried out by the company’s instructions.

Tubes without anticoagulants were used for blood samples. Centrifugation was performed after coagulation and the samples were stored at −80°C until analysis. ALT and AST analysis were performed with a Cobas 8,000 autoanalyzer (Roche Diagnostics GmBH, Mannheim, Germany) using commercial kits (Roche Diagnostics).

### 2.6 Histopathological examination

Liver tissues were first defined in a 10% formaldehyde solution. The samples were then washed in tap water for 24 h. The water in the samples was removed by passing through the alcohol series. Dehydrated tissues were treated with xylol and embedded in paraffin. Sections of 4–5 microns were obtained from paraffin blocks. It was then stained with hematoxylin-eosin. After evaluation with a light microscope, the tissues were photographed (Olympus^®^ Inc. Tokyo, Japan). The evaluation was performed by the pathologist using blinding. Liver tissues were scored for degenerative change, necrosis, and inflammation in the portal area (0; normal, 1; mild injury, 2; moderate injury, 3; severe injury).

### 2.7 Statistical analysis

Statistical analyses were performed using the IBM SPSS Statistics for Windows, 2013, Version 22.0. The mean ± standard deviation (SD) was used for the presentation of the data. The normal distribution of the data was tested with the Shapiro-Wilk test. Since MDA, TNF-α, NF-kB, ALT, and AST were normally distributed, the analysis was performed by one-way ANOVA. According to Levene test results, Tukey test was used for MDA, TNF-α, and ALT for pairwise comparisons, and the Games-Howell test was used for NF-kB and AST. Kruskal–Wallis test was preferred because tGSH, TOS, TAS, and IL-1β were not normally distributed. Since the histopathological data were semi-quantitative, the analysis was done with the Kruskal Wallis test and then the Dunn test was applied. The results were presented as median (maximum-minimum). In comparisons, the *p* < 0.05 value was determined as significant.

## 3 Results

### 3.1 Biochemical results

#### 3.1.1 MDA and tGSH analysis results

**FIGURE 1:**
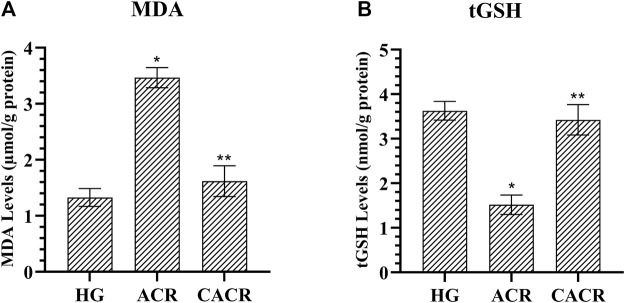
**(A,B)** Analysis of MDA and tGSH levels in liver tissues of rats in experimental groups. n = 6/each group. The bars show the mean ± standard deviation. *p < 0.05, ACR group is compared with the HG and CACR groups. **p > 0.05, CACR group is compared with the HG group.

As seen in Figure 1, acrylamide (3.47 ± 0.18) was found to significantly increase the amount of MDA compared to HG (1.33 ± 0.16) group (*p* < 0.001). MDA levels were found to be lower than ACR in the CACR group (*p* < 0.001; Figure 1). MDA levels were similar between CACR (1.62 ± 0.28) and HG (*p* = 0.073; Figure 1). In addition, acrylamide (1.52 ± 0.22) significantly reduced tGSH levels compared to HG (*p* < 0.002; Figure 1). It was determined that carvacrol significantly prevented the decrease of tGSH levels with acrylamide in liver tissue (*p* < 0.039; Figure 1). There was a similarity between the HG (3.63 ± 0.21) and CACR (3.42 ± 0.34) in terms of tGSH levels (*p* = 1.000; Figure 1).

#### 3.1.2 TOS and TAS analysis results

**FIGURE 2:**
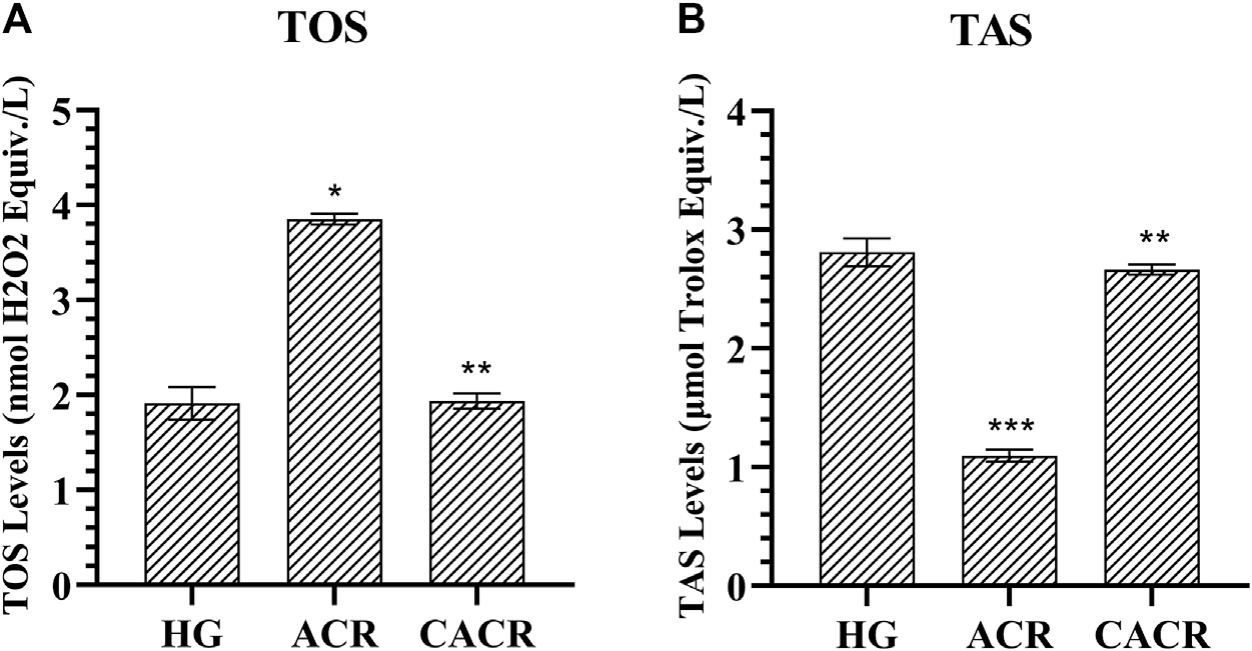
**(A,B)** Analysis of TOS and TAS levels in liver tissues of rats in experimental groups. n = 6/each group. The bars show the mean ± standard deviation. *p < 0.05, ACR group is compared with the HG and CACR groups. **p > 0.05, CACR group is compared with the HG group. ***p = 0.001, ACR group is compared with the HG group.

TOS levels in the ACR (3.85 ± 0.06) group were higher compared to CACR and HG (*p* < 0.05; Figure 2). TOS levels were similar in HG (1.91 ± 0.17) and CACR (3.42 ± 0.34) groups (*p* = 1.000; Figure 2). TAS values were lower in the ACR (1.10 ± 0.05) than in the HG (2.81 ± 0.12) group (*p* < 0.05; Figure 2). TAS levels of CACR (2.66 ± 0.04) and HG were similar (*p* = 0.479, Figure 2).

#### 3.1.3 TNF-α, IL-1β and NF-κB analysis results

**FIGURE 3:**
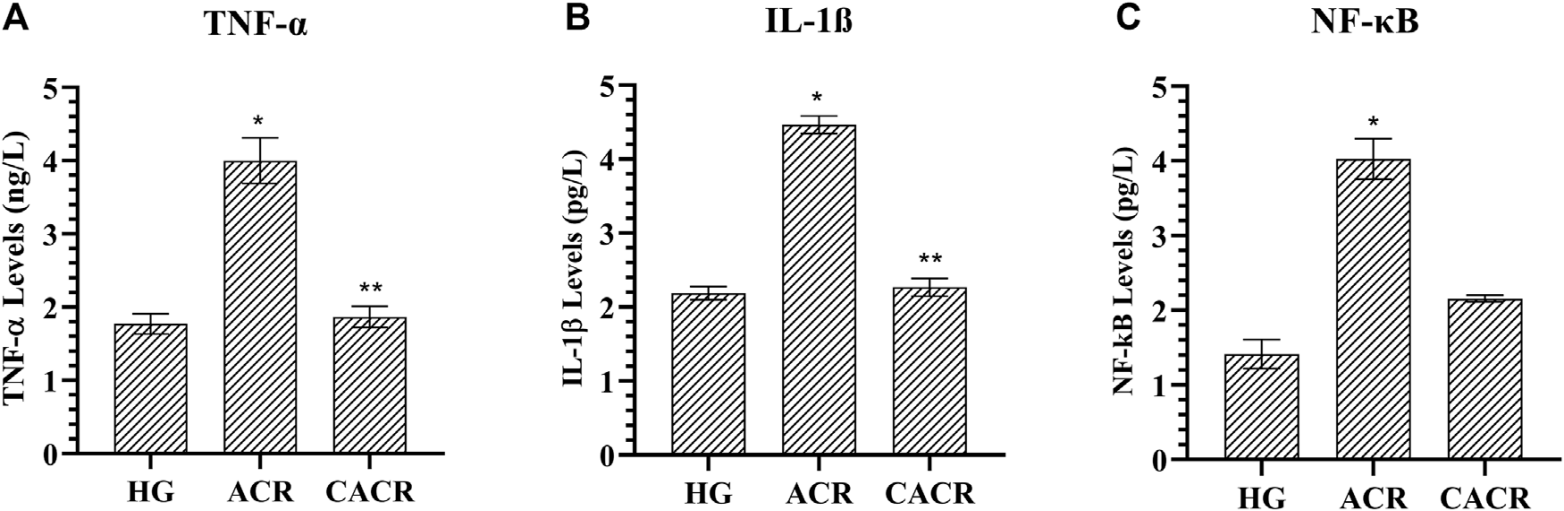
**(A–C)** Analysis of TNF-α*,* IL-1β and NF- κB levels in liver tissues of rats in experimental groups. n = 6/each group. The bars show the mean ± standard deviation. *p < 0.05, ACR group is compared with the HG and CACR groups. **p > 0.05, CACR group is compared with the HG group.

TNF-α, IL-1β, and NF-κB levels were higher in animals in the ACR (4.46 ± 0.12, 4.00 ± 0.31, 4.03 ± 0.27, respectively) than CACR (2.27 ± 0.12, 1.87 ± 0.14, 2.16 ± 0.05, respectively) and HG (2.19 ± 0.09, 1.77 ± 0.14, 0.41 ± 0.19, respectively) groups (*p* < 0.05; Figure 3). The differences between CACR and HG in terms of IL-1β and TNF-α levels were insignificant (*p* > 0.05; Figure 3).

#### 3.1.4 ALT and AST analysis results

**FIGURE 4:**
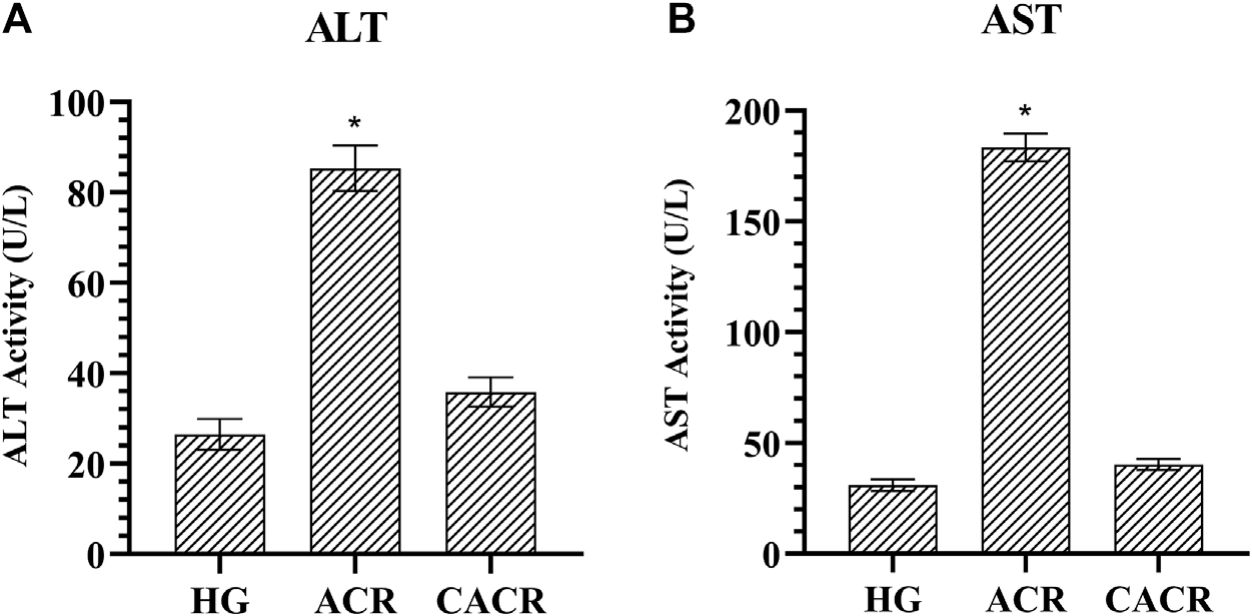
**(A,B)** Analysis of ALT and AST levels in liver tissues of rats in experimental groups. n = 6/each group. The bars show the mean ± standard deviation. *p < 0.001, ACR group is compared with the HG and CACR groups. **p > 0.05, CACR group is compared with the HG group.

Serum ALT and AST levels were higher than HG (26.58 ± 3.39, 31.00 ± 2.61, respectively) in the ACR (85.33 ± 5.01, 183.33 ± 6.22, respectively) group (*p* < 0.001; Figure 4). These values were lower in CACR (35.83 ± 3.25, 40.33 ± 2.50, respectively) than in ACR (*p* < 0.001; Figure 4). The difference between CACR group and HG was significant for ALT and AST (*p* < 0.05; Figure 4).

### 3.2 Histopathological findings

**FIGURE 5:**
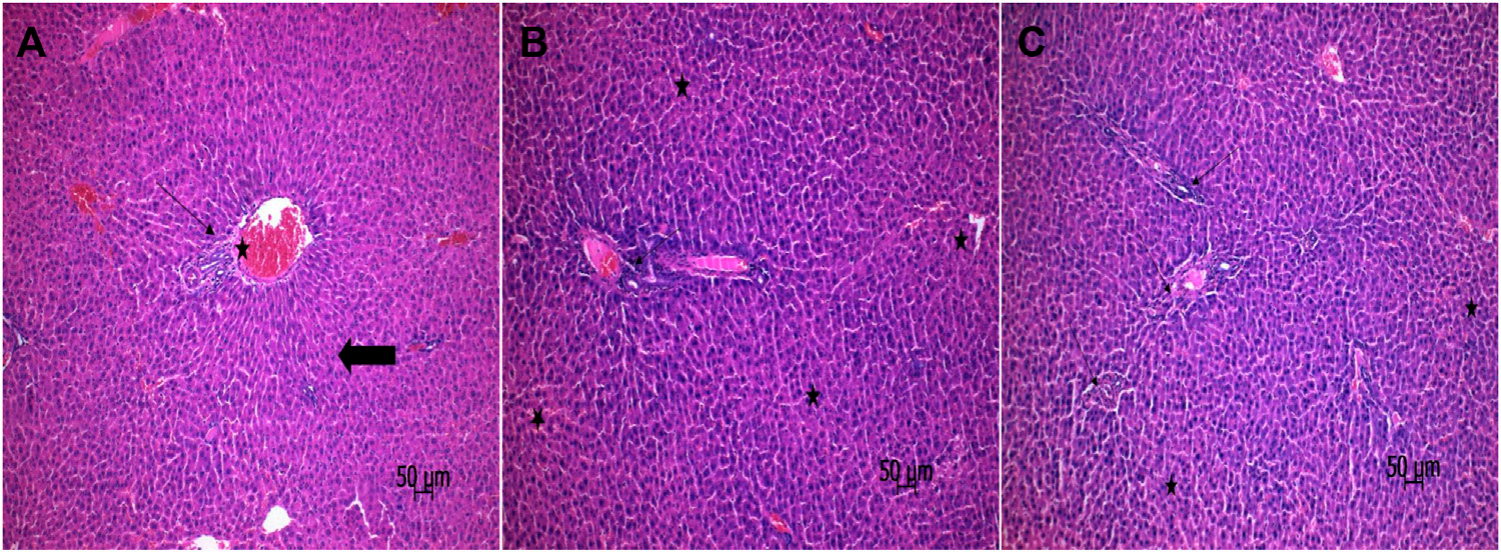
**(A–C) (A)** Sections of the normal liver tissue of the HG. Normal portal area (star), parenchyma (thick arrow) and limiting plate (thin arrow) appearance in liver tissue (H&E) **(B)** Histopathological image of ACR group. Diffuse degenerative changes and necrosis (star) in hepatocytes and moderate inflammation (thin arrow) in the portal area of the liver tissue. (H&E) **(C)** Histopathological image of the CACR group. Mild focal degenerative changes and necrosis (star) in hepatocytes, and mild inflammation (thin arrow) in portal areas (H&E).

The liver tissues in the healthy group were normal histologically (Figure 5A). However, diffuse degenerative changes and necrosis in hepatocytes, and moderate inflammation in the portal area were detected in the liver tissue of the ACR group (Figure 5B). Mild focal degenerative changes and necrosis, and mild inflammation in the portal areas were detected in the hepatocytes of the CACR group (Figure 5C). While there was a statistically significant difference (*p* < 0.001) between the ACR (3(2-3), 3(2-3), 2(2-3), respectively) group and the HG (0 (0-0), for each scoring) group in the histopathological scoring of the tissues in terms of degenerative change, necrosis and inflammation, the data of the HG and CACR (1 (1-2), for each scoring) groups were similar (*p* > 0.05).

## 4 Discussion

In this study, biochemical and histopathological examinations were performed to see the effect of carvacrol on acrylamide-induced liver toxicity in rats. Our biochemical test results showed that acrylamide increased the levels of MDA, TOS, TNF-α, IL-1β, and NF- κB decreased the levels of tGSH and TAS in liver tissue. It was also observed that acrylamide caused histopathological damage. It was determined that biochemical changes and histopathological damage were significantly prevented by carvacrol administration.

Many studies have reported that acrylamide causes an increase in the production of ROS in the liver tissue, disrupting the antioxidant balance and ultimately leading to oxidative stress (Bo et al., 2020; Rahbardar et al., 2021). In addition, it is known that ROS oxidizes the lipids in the cell membrane, disrupts the integrity of the membrane, and causes the formation of toxic products such as MDA (Li et al., 2015). Many studies are reporting that acrylamide increases MDA levels by causing lipid peroxidation (Sadek, 2012; Ybañez-Julca et al., 2022). In our study, the MDA elevation in the acrylamide group supports the literature. In addition, TOS measurement was performed to evaluate other oxidant levels in our study. It was found that high MDA levels in the acrylamide group were accompanied by high TOS levels. As is known, there is a wide spectrum of oxidant molecules. Measurements of these oxidants separately increase the cost. Therefore, all of the ROSs are currently being investigated by TOS analysis (Erel, 2005). In a study by Gedik et al., it was reported that TOS level was also increased in damaged liver tissue belonging to the acrylamide group in which MDA was measured high (Gedik et al., 2017). There is no data in the literature on whether carvacrol protects liver tissue from acrylamide-induced oxidative damage. However, Bozkurt et al. reported that carvacrol had a hepatoprotective effect by suppressing the increase of MDA and TOS levels in rat liver tissue with methotrexate (Bozkurt et al., 2014). There is also information in the literature showing that carvacrol prevents liver damage by reducing the severity of lipid peroxidation reaction (Baranauskaite et al., 2020). Our study findings and the information obtained from previous studies reveal that carvacrol shows antioxidant activity.

In our study, a decrease in the level of tGSH, an important endogenous antioxidant, was detected in the liver tissue, along with an increase in MDA and TOS in the acrylamide group. GSH is a low molecular weight thiol compound found in living tissues. GSH protects cells against ROS damage by maintaining the cell’s redox state (Ulrich and Jakob, 2019). There are studies documenting a decrease in the amount of GSH in acrylamide-induced liver oxidative damage (Zhao et al., 2015; Rahbardar et al., 2021). There are no studies investigating the effect of acrylamide on tGSH in liver tissue. However, it has been reported that acrylamide causes a decrease in tGSH levels by increasing oxidative stress in the kidney, brain, and intestinal tissues (Yang et al., 2019; Bedir et al., 2021; Turan et al., 2021). In our study, liver TAS level was measured to evaluate the effect of acrylamide on other antioxidants besides tGSH. TAS is used to measure the cumulative antioxidative effects of all antioxidants in organisms (Erel, 2004a). In our study, it was determined that the TAS level decreased in parallel with the tGSH level in the acrylamide group. This suggests that in the acrylamide group, antioxidant systems are insufficient to neutralize ROS. Gursul et al. reported that carvacrol protected the liver tissue from oxidative damage by preventing the decrease in tGSH and TAS (Gursul et al., 2022). In another study, it was reported that carvacrol protects liver tissue against oxidative stress (Imtara et al., 2021). This information indicates that carvacrol has antioxidant properties in liver tissue.

Many experimental studies have reported that excessive production of ROS in liver damage causes excessive production of proinflammatory cytokines in cells (Pizzino et al., 2017; Pomacu et al., 2021). MAPK and NF-κB signaling pathways are activated when cellular stress and ROS production increase (Hong et al., 2019). NF- κB, a transcription factor, accelerates the inflammatory process by triggering the release of proinflammatory cytokines (Liu et al., 2017). In our study, the increase in TNF-α and IL-1β levels together with NF-κB in liver tissue with acrylamide may be due to the induction of proinflammatory cytokine synthesis by NF-κB. In a study by Dönmez et al., it was reported that acrylamide significantly induced NF- κB expression in liver tissue (Donmez et al., 2020). In addition, Yang et al. suggested that there was an increase in TNF-α and IL-1β secretion in liver tissue due to the activation of the NF- κB signaling pathway in the inflammatory process caused by acrylamide (Nan et al., 2021). The lower, TNF-α, IL-1β, and NF- κB levels in the carvacrol group compared to the acrylamide group indicate that it antagonizes acrylamide-related hepatoinflammation. Kandemir et al. showed that carvacrol ameliorates inflammation in hepatic tissue by inhibiting the increase of inflammatory cytokines (Kandemir et al., 2021).

Changes in the activities of serum ALT and AST enzymes reflect damage to liver cells (Bo et al., 2020; Rahbardar et al., 2021). Acrylamide-induced ROS causes the degradation of polyunsaturated fatty acids in the structure of the cell membrane. This disrupts the structure of the cell membrane and subsequently leads to significant elevations of serum ALT and AST levels (Erfan et al., 2021). Our study determined that rats treated with acrylamide due to liver damage had increased serum ALT and AST levels. Elevated serum ALT and AST levels are probably due to the leakage of these enzymes into the circulation as a result of cell membrane damage caused by oxidative stress (Gedik et al., 2017; Ybañez-Julca et al., 2022). We found that ALT and AST levels approached normal values as a result of carvacrol administration. Studies in the literature support our experimental results, that carvacrol prevents the increase in serum ALT and AST levels and alleviates liver damage (Bakır et al., 2016; Mohebbati et al., 2018).

In the current study, it was seen that the histological analysis results of the tissues were compatible with the biochemical data. While mild degeneration was observed in liver hepatocytes of animals treated with carvacrol, by previous studies (Gedik et al., 2017; Kandemir et al., 2021), inflammatory cell infiltration was observed in the portal region of the liver tissue of rats in the acrylamide group. In the study of Karimi et al., which supports our histopathological findings, inflammatory cell infiltration was found in the liver tissue of the animals treated with acrylamide (Karimi et al., 2020). This acrylamide-related histopathological damage has been associated with an increase in oxidant and proinflammatory cytokines. Also, hepatocellular necrosis of different sizes and diffuse degenerative changes were detected in the liver parenchyma of the animals to which we administered acrylamide. In another previous study, acrylamide was documented to cause severe damage such as hepatocyte degeneration, inflammation, congestion, necrosis, and fibrosis in rat liver injury (Cengiz et al., 2022).

## 5 Conclusion

In the present study, oxidative and inflammatory damage developed in acrylamide-treated liver tissues of rats. Our findings indicate that carvacrol has a hepatoprotective effect by suppressing the increase in MDA, TOS, TNF-α, IL-1β, NF- κB, ALT, and AST levels with acrylamide, and the decrease in tGSH and TAS levels. These results suggest that carvacrol may be useful in the treatment of acrylamide-induced hepatotoxicity.

## 6 Limitations

Further studies should be conducted for molecular histopathological examination of acrylamide-induced liver injury.

## Data Availability

The original contributions presented in the study are included in the article/supplementary materials, further inquiries can be directed to the corresponding author.
